# Phosphate-Based Ultrahigh Molecular Weight Polyethylene Fibers for Efficient Removal of Uranium from Carbonate Solution Containing Fluoride Ions

**DOI:** 10.3390/molecules23061245

**Published:** 2018-05-23

**Authors:** Rong Li, Yuna Li, Maojiang Zhang, Zhe Xing, Hongjuan Ma, Guozhong Wu

**Affiliations:** 1 Shanghai Institute of Applied Physics, Chinese Academy of Sciences, Shanghai 201800, China; lirong@sinap.ac.cn (R.L.); liyuna@sinap.ac.cn (Y.L.); zhangmaojiang@sinap.ac.cn (M.Z.); xingzhe@sinap.ac.cn (Z.X.); mahongjuan@sinap.ac.cn (H.M.); 2 Department of Chemistry, College of Sciences, Shanghai University, Shanghai 200444, China; 3 University of Chinese Academy of Sciences, Beijing 100049, China

**Keywords:** ultrahigh molecular weight polyethylene fibers, radiation induced graft polymerization, glycidyl methacrylate, phosphate group, removal of uranium from carbonate solution

## Abstract

This work provides a cost-effective approach for preparing functional polymeric fibers used for removing uranium (U(VI)) from carbonate solution containing NaF. Phosphate-based ultrahigh molecular weight polyethylene (UHMWPE-g-PO_4_) fibers were developed by grafting of glycidyl methacrylate, and ring-opening reaction using phosphoric acid. Uranium (U(VI)) adsorption capacity of UHMWPE-g-PO_4_ fibers was dependent on the density of phosphate groups (D_PO_, mmol∙g^−1^). UHMWPE-g-PO_4_ fibers with a D_PO_ of 2.01 mmol∙g^−1^ removed 99.5% of U(VI) from a Na_2_CO_3_ solution without the presence of NaF. In addition, when NaF concentration was 3 g∙L^−1^, 150 times larger than that of U(VI), the U(VI) removal ratio was still able to reach 92%. The adsorption process was proved to follow pseudo-second-order kinetics and Langmuir isotherm model. The experimental maximum U(VI) adsorption capacity (Q_max_) of UHMWPE-g-PO_4_ fibers reached 110.7 mg∙g^−1^, which is close to the calculated Q_max_ (117.1 mg∙g^−1^) by Langmuir equation. Compared to F^−^, Cl^−^, NO_3_^−^, and SO_4_^2^^−^ did not influence U(VI) removal ratio, but, H_2_PO_4_^−^ and CO_3_^2^^−^ significantly reduced U(VI) removal ratio in the order of F^−^ > H_2_PO_4_^−^ > CO_3_^2^^−^. Cyclic U(VI) sorption-desorption tests suggested that UHMWPE-g-PO_4_ fibers were reusable. These results support that UHMWPE-g-PO_4_ fibers can efficiently remove U(VI) from carbonate solutions containing NaF.

## 1. Introduction

During the process of uranium enrichment, the yellow cake is converted into uranium hexafluoride (UF_6_) gas for uranium isotopic enrichment, accompanied by the generation of exhaust gas, which is treated with aqueous sodium carbonate (Na_2_CO_3_) solution. Consequently, an alkaline uranium-rich effluent is generated, where uranium exists mainly as uranyl carbonate complexes, e.g., UO_2_(CO_3_)_2_^2^^−^ and UO_2_(CO_3_)_3_^4^^−^ [1,2]. The efficient sequestration of uranium from its secondary sources and wastewater, to decrease the uranium concentration below the recommended value for discharge, is one of the biggest challenges of the uranium enrichment industry, and developing techniques to solve this problem has attracted great interest [3]. This is mainly driven by two factors: (1) reducing uranium pollution to protect the environment, ecosystem, and human health, and (2) recycling and saving uranium resources [4].

Several main methods for the recovery of uranium from aqueous solution have been investigated over the past decades, including solvent extraction, ion exchange, and adsorption. Solvent extraction, as a well-established method, is economically viable when the concentration of solute and the flow rate of wastewater are both high, and becomes uneconomic when the concentration of solute is lower than 0.5 g∙L^−1^ [5]. Additionally, this method is to some degree not environment-friendly. As a well-known example of solvent extraction operation, the plutonium uranium recovery by extraction (PUREX) process, used for recovering uranium and plutonium, produces large amounts of aqueous and organic radioactive waste solutions [6]. For the ion-exchange technology, the volume of ion-exchange resin used is dependent on the concentration of solute. Thus, when the concentration of solute is high, a large size of equipment is necessary, which makes such a process economically unfeasible [5]. Research has therefore been mainly focused on developing functional solid sorbents with selectivity. Because adsorption techniques have significant merits, including good feasibility and practicality, and flexible design and operation, various kinds of sorbents have been fabricated for the extraction of uranium from water solutions, e.g., synthetic polymeric [7], biopolymeric [8], inorganic [9], mesoporous silica-based [10], porous carbon-based [11], metal−organic framework-based [12], and ionic liquids [13] adsorbents. Among these types of sorbents, the polymeric sorbents, especially polymeric fibers, have several advantages, such as light weight, simple process of fabrication into various shapes and lengths, facility of deployment, and ease of recyclability and reusability [14]. 

The amidoxime group has been proven to have a high affinity for uranium in aqueous solutions. Amidoxime-based polymeric fibers are extensively used for the capture of uranium from aqueous solution and seawater [15]. However, amidoxime-based sorbents suffer from one main shortcoming, i.e., relatively slow sorption kinetics, which has been attributed to a reaction-limited process [16]. Additionally, the acrylonitrile monomer used to prepare amidoxime-based sorbents is explosive and toxic. Adsorbents with phosphonic acid functionality are widely used for the extraction and the separation of lanthanides and actinides, since phosphonic acid groups can form stable complexes with them [17]. Research has been conducted on the recovery of uranium species from aqueous phases by sorbent-tethered phosphonic acid groups, e.g., phosphonic acid-based mesoporous silica [17,18] and poly(styrene-co-divinylbenzene) [19], vinylphosphonic acid grafted poly(vinyl alcohol) fibers [20], phosphate-based mesoporous carbon [21], and poly(ethylene glycol methacrylate phosphate) grafted polypropylene membrane [22]. However, it is difficult to find a technically and economically feasible sorbent, fabricated in a simple way using inexpensive precursors, that can be considered as a potential sorbent for recovering uranium from wastewater.

**Scheme 1:**
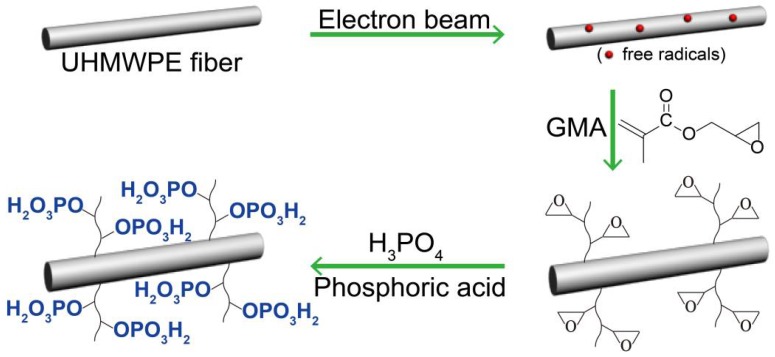
The synthetic route for UHMWPE-g-PO_4_ fiber sorbent.

Herein, we developed a kind of phosphate-based ultrahigh molecular weight polyethylene (UHMWPE-g-PO_4_) fiber adsorbent by the radiation grafting of glycidyl methacrylate (GMA) and ring-opening reaction of epoxy groups with phosphoric acid (H_3_PO_4_) (see Scheme 1). UHMWPE fiber was used as the substrate, owing to its property of high strength and excellent resistance to corrosion even after radiation grafting [23]. Moreover, the free radicals formed in the UHMWPE fiber have a long life span, which is beneficial for the grafting reaction [24]. In this work, the uranium (U(VI)) loading capacity and removal ratio of the UHMWPE-g-PO_4_ fiber sorbent were evaluated by adsorption experiments performed in solution, prepared with uranyl nitrate hexahydrate (UO_2_(NO_3_)_2_·6H_2_O), Na_2_CO_3_, sodium fluoride (NaF), and deionized water. The molar ratio of UO_2_(NO_3_)_2_·6H_2_O to anhydrous Na_2_CO_3_ was 1:5 in all the U(VI) aqueous solutions. In previous works, anionic resins [1,25] and ionic liquid [13] have been used to extract U(VI) from carbonate solution containing fluoride ions. To the best of our knowledge, this research is the first reported work on phosphate-based sorbents used for removal of U(VI) from carbonate solution containing fluoride ions.

## 2. Results

### 2.1. Radiation Grafting Kinetics of GMA

**Figure 1:**
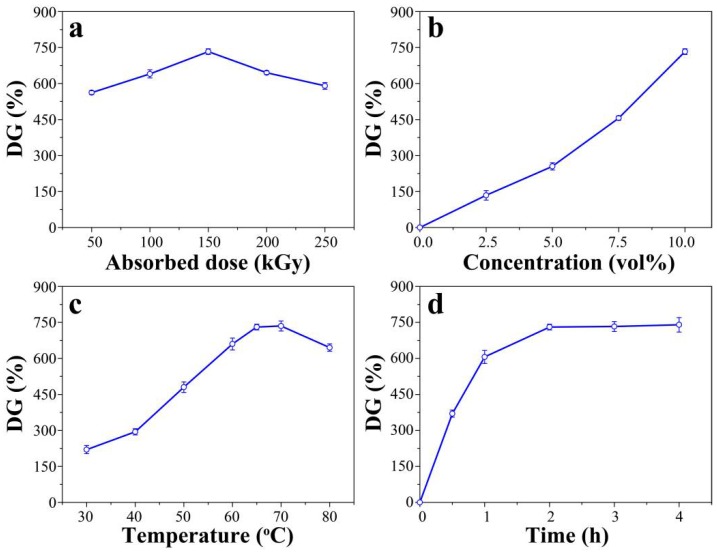
DG of UHMWPE fibers as a function of (**a**) absorbed dose (10 vol%, 65 °C, 2 h), (**b**) GMA concentration (150 kGy, 65 °C, 2 h), (**c**) reaction temperature (150 kGy, 10 vol%, 2 h), and (**d**) reaction time (150 kGy, 10vol%, 65 °C).

The kinetics of graft polymerization of GMA onto the UHMWPE fibers was investigated to determine the optimum grafting parameters. Figure 1a shows the degree of grafting (DG) versus the absorbed dose. The DG initially enhances with an increase of absorbed dose from 50 to 150 kGy attributable to the increasing absorbed dose raising the amount of free radicals [24]. However, the DG declines with a further increase of the absorbed dose from 150 to 250 kGy. This is mainly ascribed to radiation-induced degradation of the UHMWPE chains from the high absorbed dose in air [26]. Figure 1b describes the relationship between DG and monomer concentration. As anticipated, the DG logically increases with the GMA concentration due to more available monomers taking part in the graft reaction. Figure 1c depicts the effect of reaction temperature on the DG. An increase in the temperature from 30 to 70 °C is accompanied by an increase in the DG. This is ascribed to the high temperature stimulating the diffusion of monomer into the grafting sites on fibers [27]. However, the DG reduces when the temperature is more than 70 °C. This might be caused by GMA homopolymerization dominating at high temperature. Figure 1d portrays the influence of reaction time on the DG. The DG increases for the first 2 h, and then tends to level off. Consequently, the optimum grafting reaction conditions might be 150 kGy, 65–70 °C, and 2 h, and the desired DG can be simply obtained by adjusting the monomer concentration.

### 2.2. Characterization of Modified UHMWPE Fibers

**Figure 2:**
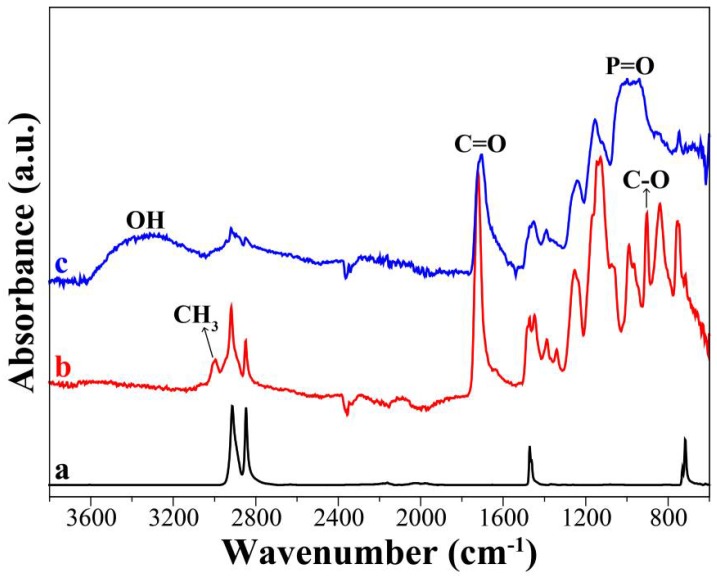
ATR-FTIR spectroscopy of (**a**) original UHMWPE, (**b**) UHMWPE-g-PGMA (DG = 540%), and (**c**) UHMWPE-PO_4_ (D_PO_ = 1.93 mmol∙g^−1^) fibers.

Figure 2 portrays the attenuated total reflectance Fourier transform infrared (ATR-FTIR) spectroscopy of UHMWPE fibers. The original UHMWPE fiber has reflection bands at 2909, 2843, 1468, and 715 cm^−1^, which are due to the asymmetrical stretching, symmetrical stretching, bending, and rocking vibrations of methylene (CH_2_), respectively. After grafting (trace b), the stretching vibrations of CH_3_ at 2918 cm^−1^, C=O at 1720 cm^−1^, and C–O (ring) at 905 cm^−1^ confirm the successful grafting of GMA onto UHMWPE fiber [28]. After phosphation (trace c), the absorption peak of C–O (ring) disappears, and fresh peaks of OH and P=O originate at 3100–3500 and at 930–1025 cm^−1^, respectively, demonstrating the successful introduction of the phosphate group onto UHMWPE fiber [29]. 

**Figure 3:**
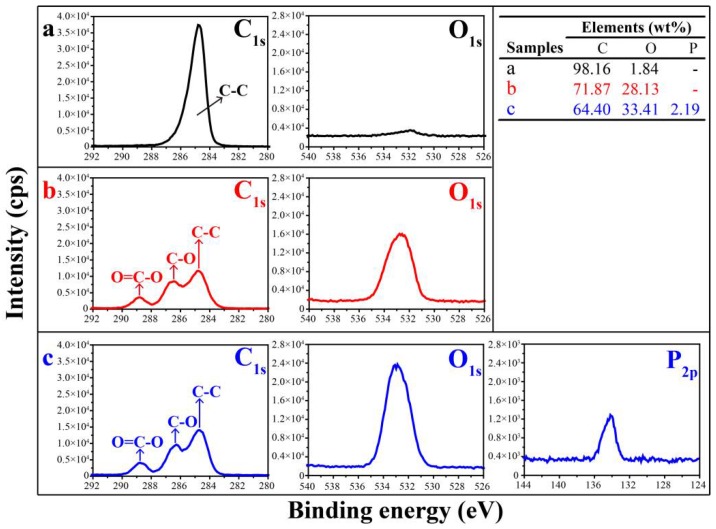
XPS spectra and the elements (inserted Table) of (**a**) pristine UHMWPE, (**b**) UHMWPE-g-PGMA (DG = 540%), and (**c**) UHMWPE-g-PO_4_ (D_PO_ = 1.93 mmol∙g^−1^) fibers.

The chemical composition of the UHMWPE fibers was analyzed by X-ray photoelectron spectroscopy (XPS), as portrayed in Figure 3. The pristine UHMWPE fiber exhibits a strong C1s peak at 284.8 eV (C–C) and a weak O1s peak at 531.9 eV. The emergence of oxygen (O, 1.84%) element in the pristine UHMWPE fiber might be due to oxidation or contamination. After grafting of GMA, the O content evidently increases from 1.84% to 28.13%, combined with a strong O1s peak at 532.8 eV. The C content decreases from 98.16% to 71.87%, and the C1s peak can be clearly decomposed into C–C (284.8 eV), C–O (286.5 eV), and O=C–O (288.8 eV) peaks. For the UHMWPE-g-PO_4_ fiber, the C content decreases from 71.87% to 64.40%, and the O content increases from 28.13% to 33.14%, coupled with a much stronger O1s peak. Moreover, the appearance of a novel P_2p_ peak at 134.0 eV illustrates that phosphate groups are successfully introduced into the UHMWPE fibers.

### 2.3. Uranium Adsorption

Screening tests were conducted in U(VI) carbonate solutions with NaF to identify sorbents with high U(VI) adsorption capacities. As shown in Table 1, among the tested sorbents the sorbent with the highest D_PO_ presents the highest U(VI) adsorption capacity.

**Figure 4:**
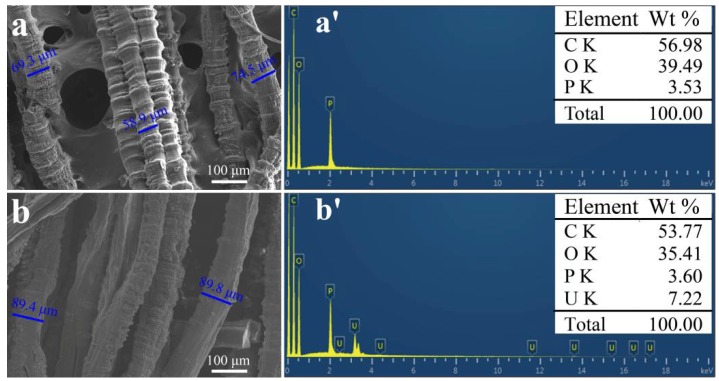
SEM pictures, EDX spectra of UHMWPE-g-PO_4_ fibers (DG = 630%; D_PO_ = 2.01 mmol∙g^−1^) before (**a**,**a′**) and after (**b**,**b′**) U(VI) adsorption (C_0_: 20.0 mg∙L^−1^, sorbent: 0.2 g, volume: 1 L, NaF: 2.0 g∙L^−1^, time: 24 h, and temperature: 25 °C).

In order to compare the surface morphologies and elemental contents of the UHMWPE-g-PO_4_ fibers before and after U(VI) adsorption, a scanning electron microscope (SEM) equipped with energy dispersive X-ray (EDX) spectroscopy was used to observe the sample surface. Figure 4 displays the surface morphologies and EDX spectra of the non-adsorbed and U(VI)-loaded UHMWPE-g-PO_4_ fibers. Comparing Figure 4a with Figure 4b, it can be seen that the loaded U(VI) increases the mean diameter of the sorbent from ~67.6 μm to ~89.6 μm. The EDX spectra and elemental contents are depicted in Figure 4a′,b′, and the inserted tables, respectively. In comparison with non-adsorbed fiber, the U(VI)-loaded fiber presents obvious U(VI) absorption peaks in Figure 4b′, and the content of U(VI) (7.22 wt%) in the sorbent, to a certain extent, indicates the UHMWPE-g-PO_4_ sorbent can effectively capture uranium from carbonate solution with NaF.

**Figure 5:**
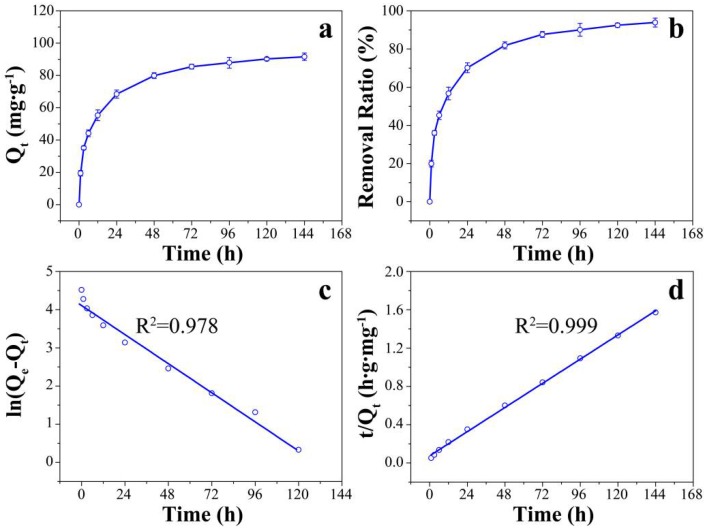
Influence of sorption time on the (**a**) U(VI) adsorption capacity and (**b**) removal ratio. U(VI) adsorption kinetic curves: (**c**) linearized pseudo-first-order and (**d**) pseudo-second-order kinetic models (C_0_: 19.7 mg∙L^−1^, sorbent: 0.2 g, volume: 1 L, NaF: 2.0 g∙L^−1^, and temperature: 25 °C).

The effect of sorption time on the U(VI) loading capacity and removal ratio of UHMWPE-g-PO_4_ fibers (D_PO_ = 2.01 mmol∙g^−1^) was investigated using batch experiments at various time intervals from 0 to 144 h, in order to determine the sorption kinetics. As shown in Figure 5a,b, both U(VI) adsorption capacity and removal ratio increase significantly with the sorption time during the first 24 h, rise gradually up to 72 h, and then level off to an equilibrium state. The U(VI) adsorption capacity and removal ratio reach 68.5 mg∙g^−1^ and 70% within 24 h, and 91.6 mg∙g^−1^ and 93% at equilibrium, respectively. 

Additionally, in order to understand the adsorption kinetics, pseudo-first-order and pseudo-second-order models were used to fit the experimental data. The two kinetic models were given in the linear form:

pseudo-first-order model: ln (Q_e_ − Q_t_) = lnQ_e_ − *k*_1_t (1)

pseudo-second-order model: t/Q_t_ = 1/(*k*_2_∙Q_e_^2^) + t/Q_e_ (2)

where Q_e_ (mg∙g^−1^) and Q_t_ (mg∙g^−1^) are the U(VI) loading amounts at equilibrium and at various contact time “t”, respectively. *k*_1_ (h^−1^) and *k*_2_ (g∙mg^−1^∙h^−1^) are the pseudo-first-order and the pseudo-second-order rate constants of adsorption, respectively. 

Two straight lines with correlation coefficients (R^2^) acquired by linear regression are shown in Figure 5c (pseudo-first-order) and Figure 5d (pseudo-second-order), respectively. The values of Q_e_, *k*_1_, *k*_2_, and R^2^ are summarized in Table 2. The value of R^2^ (0.999) for the pseudo-second-order model is higher than that (0.978) of the pseudo-first-order model. Furthermore, as compared with that (63.1 mg∙g^−1^) of the pseudo-first-order model, the calculated Q_e_ (95.2 mg∙g^−1^) by the pseudo-second-order model is almost equal to that of the experimental Q_e_ (91.6 mg∙g^−1^). As a result, it can be concluded that the U(VI) adsorption kinetics on the UHMWPE-g-PO_4_ fiber follow a pseudo-second-order kinetic model.

**Figure 6:**
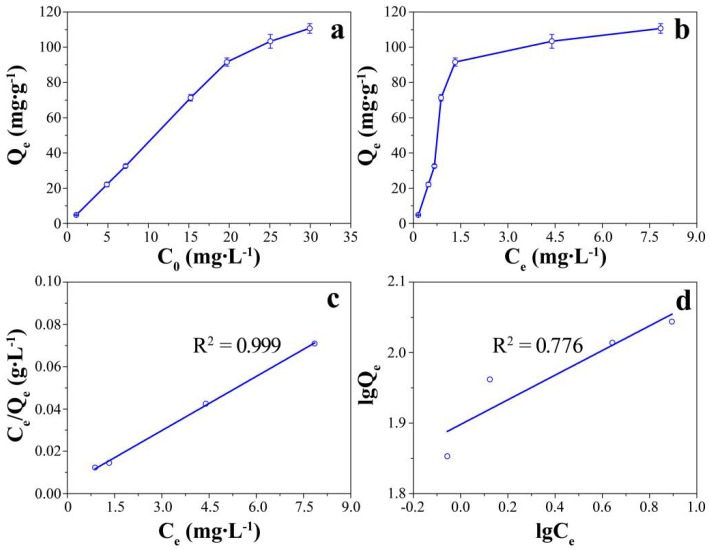
(**a**) Relationship between the Q_e_ and the initial U(VI) concentration, (**b**) U(VI) adsorption isotherm for UHMWPE-g-PO_4_ fibers, (**c**) linearized Langmuir, and (**d**) Freundlich adsorption isotherms by fitting the experimental data (sorbent: 0.2 g, volume: 1 L, time: 144 h, temperature: 25 °C, and NaF: 2 g∙L^−1^).

To assess the overall U(VI) adsorption capacity, an adsorption isotherm was collected by equilibrating the UHMWPE-g-PO_4_ fibers with a wide range of initial U(VI) concentrations. Figure 6a,b show that Q_e_ increases sharply from 4.8 mg∙g^−1^ (C_0_: 1.1 mg∙L^−1^; U(VI) concentration at adsorption equilibrium (C_e_): 0.2 mg∙L^−1^) to 91.6 mg∙g^−1^ (C_0_: 19.7 mg∙L^−1^; C_e_: 1.3 mg∙L^−1^), and then increases slowly up to 110.7 mg∙g^−1^ (C_0_: 29.9 mg∙L^−1^; C_e_, 7.8 mg∙L^−1^).

The Langmuir and Freundlich models were used to analyze the equilibrium adsorption isotherms and investigate the adsorption mechanism of the adsorbent. The two equations were listed as:

Langmuir equation: C_e_/Q_e_ = C_e_/Q_max_ + 1/(Q_max_ × *K*_L_) (3)

Freundlich equation: lgQe = lg*K*_F_ + (1/*n*) × lgC_e_ (4)

where C_e_ (mg∙L^−1^) is the U(VI) concentration at adsorption equilibrium, Q_e_ (mg∙g^−1^) is the U(VI) loading amount at equilibrium, Q_max_ is the is the saturated Langmuir monolayer sorption capacity (mg∙g^−1^), *K*_L_ (L∙mg^−1^) is the Langmuir equilibrium constant related to the energy of adsorption and affinity of the adsorbent, and *K*_F_ (mg∙g^−1^) and *n* are the Freundlich constants representing sorption capacity and sorption intensity, respectively.

The linearized plots of the Langmuir and the Freundlich adsorption isotherms are given in Figure 6c,d. The values of Q_max_, *K*_L_, *K*_F_, *n*, and R^2^ are shown in Table 3. In comparison with the *K*_F_ (79.1 mg∙g^−1^) computed by the Freundlich equation, the calculated Q_max_ (117.1 mg∙g^−1^) using the Langmuir equation is very close to the experimental Q_e_ (110.7 mg∙g^−1^) at the initial concentration of 29.9 mg∙L^−1^. Additionally, the correlation coefficient R^2^ (0.999) of the Langmuir model is higher than that (0.776) of the Freundlich model. Thus, we can conclude that the adsorption experimental results for the UHMWPE-g-PO_4_ fibers are in good agreement with the Langmuir model.

**Figure 7:**
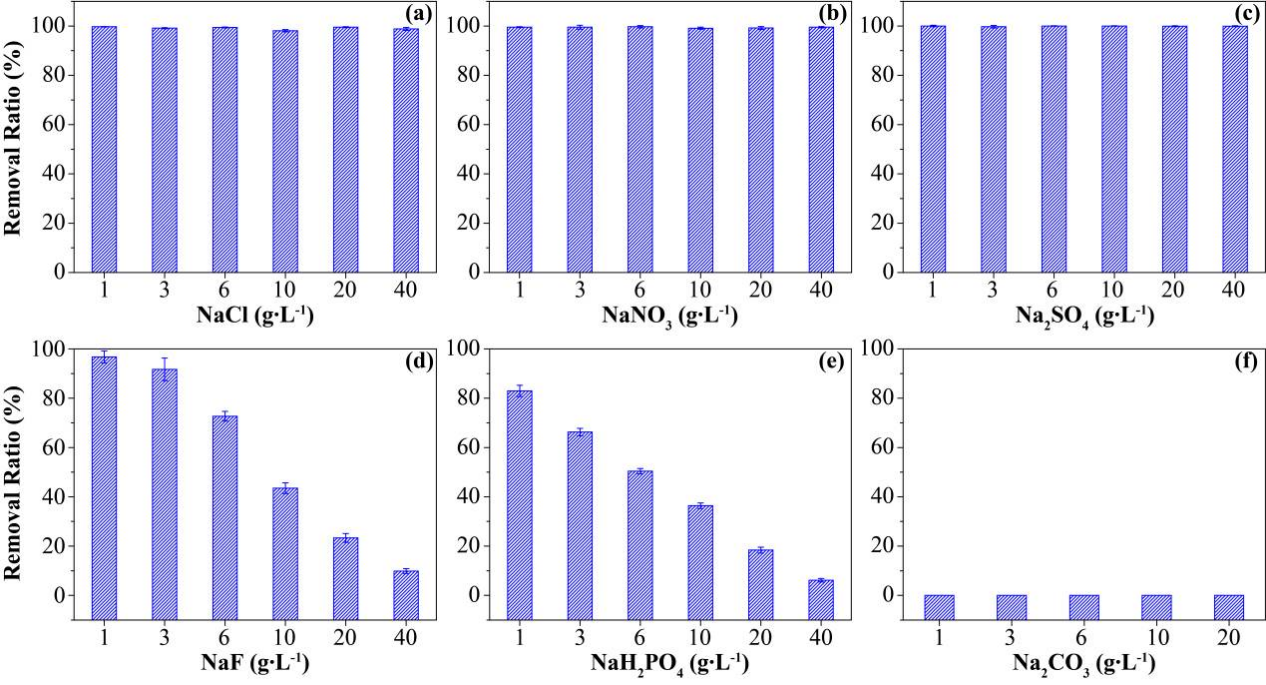
U(VI) removal ratio versus the concentrations of (**a**) NaCl, (**b**) NaNO_3_, (**c**) Na_2_SO_4_, (**d**) NaF, (**e**) NaH_2_PO_4_, and (**f**) Na_2_CO_3_ (C_0_: 19.5 mg∙L^−1^, sorbent: 0.2 g, volume: 1 L, time: 144 h, and temperature: 25 °C).

In the alkaline wastewater that is produced in a uranium enrichment plant, there is a lot of fluoride. Hence, the effect of F^−^ ions on the U(VI) removal ratio was investigated in this work. The influences of other competitive anions, including chloride (Cl^−^), nitrate (NO_3_^−^), sulfate (SO_4_^2^^−^), dihydrogen phosphate (H_2_PO_4_^−^), and CO_3_^2^^−^ on the U(VI) removal ratio were explored as well. Figure 7 shows the relationships between the concentrations of various salts and the U(VI) removal ratio of UHMWPE-g-PO_4_ fibers. It should be mentioned here that the U(VI) removal ratio is ~99.5% when no other anion is dissolved in the solution, which is used as the control. Figure 7a–c illustrate that the U(VI) removal ratios are almost unchanged with increasing concentrations of sodium chloride (NaCl), sodium nitrate (NaNO_3_), and sodium sulfate (Na_2_SO_4_). This means that Cl^−^, NO_3_^−^, and SO_4_^2^^−^ do not exhibit competitive effects during the process of U(VI) adsorption. Figure 7d portrays the effect of NaF concentration on the U(VI) removal ratio. When the NaF concentration is less than 3 g∙L^−1^, the removal ratio is higher than 92%. It should be emphasized that this NaF concentration (3 g∙L^−1^) is 150 times higher than that (~20 mg∙L^−1^) of U(VI) in the solution. This indicates that the UHMWPE-g-PO_4_ fiber sorbent has a high adsorption efficiency for U(VI) at a relatively low NaF concentration. However, with a further increase of NaF concentration from 6 to 40 g∙L^−1^, the U(VI) removal ratio declines significantly from 73% to 10%. This can be attributed to competitive coordination of U(VI) between phosphonyl oxygen and F^−^. The increasing concentration of NaF enhances the interaction between F^−^ and U(VI), thereby decreasing the interaction between phosphonyl oxygen and U(VI), and thus reducing the U(VI) removal ratio [30]. Figure 7e describes the U(VI) removal ratio as a function of sodium dihydrogen phosphate (NaH_2_PO_4_) concentration. The U(VI) removal ratio decreases with an increasing concentration of NaH_2_PO_4_. It can be ascribed to competitive coordination of U(VI) with phosphonyl oxygen from NaH_2_PO_4_. Figure 7f portrays the influence of Na_2_CO_3_ concentration on the U(VI) removal ratio. The U(VI) removal ratio is zero within the Na_2_CO_3_ concentration range of 1–20 g∙L^−1^. This is due to strong coordination between CO_3_^2^^−^ and U(VI), which significantly inhibits the coordination between U(VI) and phosphonyl oxygen [31]. 

**Figure 8:**
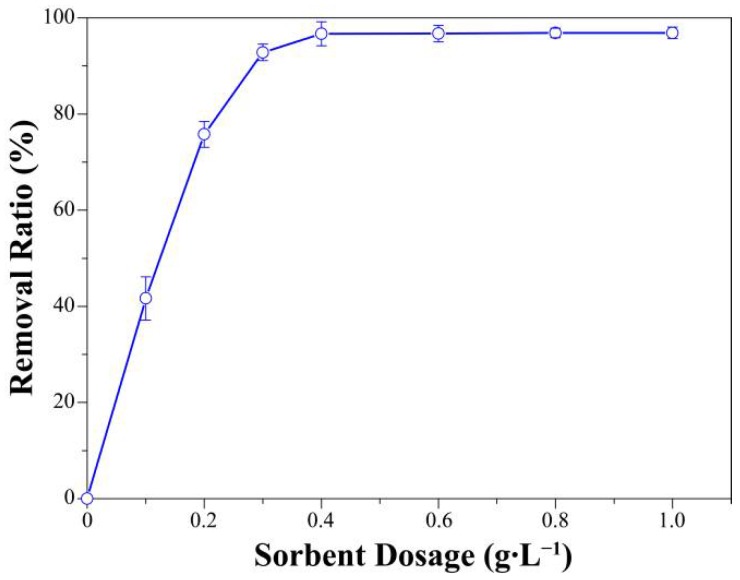
U(VI) removal ratio versus sorbent dosage (C_0_: 29.8 mg∙L^−1^, NaF: 2 g∙L^−1^, volume: 1 L, time: 144 h, and temperature: 25 °C).

From the point of view of economy, it is necessary to investigate the effect of sorbent dosage on the U(VI) removal ratio with a certain initial concentration. The influence of sorbent dosage was explored with the sorbent dosage ranging from 0.1 to 1.0 g∙L^−1^. As shown in Figure 8, the U(VI) removal ratio shows a rapid increase as the sorbent dosage is raised from 0.1 to 0.3 g∙L^−1^, owing to the higher amount of sorbent providing more available adsorption sites for capturing U(VI). At a sorbent dosage of 0.4 g∙L^−1^ or higher, the U(VI) removal ratio is invariable at 96.9%. This implies that an equilibrium has been achieved between the fibrous sorbent and the solution [32].

**Figure 9:**
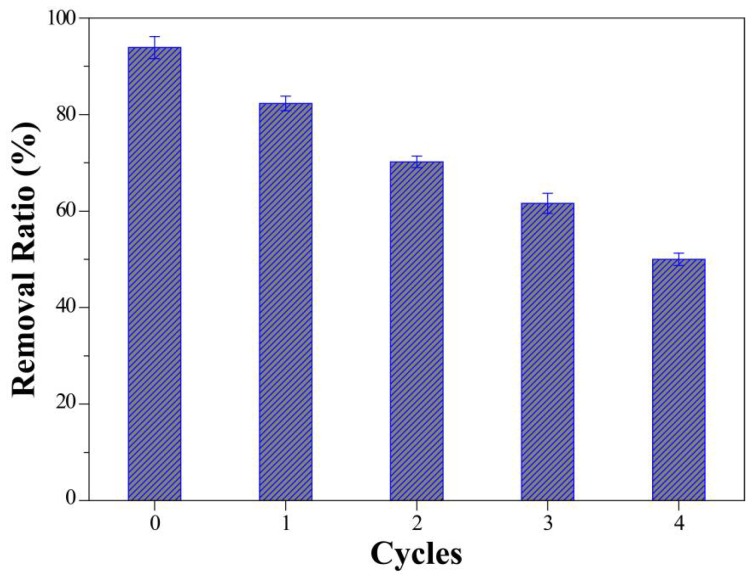
U(VI) removal ratio versus cycles (C_0_: 19.7 mg∙L^−1^, NaF: 2 g∙L^−1^, sorbent: 0.2 g, volume: 1 L, time: 144 h, and temperature: 25 °C).

Desorption of U(VI) from the UHMWPE-g-PO_4_ fibers can provide for better utilization of the fibers during the repeated recovery of U(VI), and reduce the cost of the adsorption process. Na_2_CO_3_ solution has been proved to be a good desorbent, with minimal effects on sorbent. Consequently, the Na_2_CO_3_ solution was used to desorb the loaded U(VI), and the desorbed sorbent was further used for up to four cycles of repetitive sorption-desorption under identical experimental conditions. As depicted in Figure 9, ~15% reduction in the removal ratio occurs after each consecutive run over the four cycles, thus about a 50% removal ratio was achieved in the fourth cycle. These results show the potential reusability of UHMWPE-g-PO_4_ fibers for recovering U(VI) from carbonate solution containing F^−^ ions.

## 3. Discussion

Radiation-induced graft polymerization of vinyl monomers onto polymers has received increasing attention due to its advantages of simplicity and facility to develop alternative functional polymeric materials [33]. In this work, EB irradiation was selected, owing to its high absorbed dose rate and short processing time, and was easy for pilot-scale production of functional polymers [34]. The investigation on the effects of absorbed dose, monomer concentration, temperature, and reaction time on the DG of GMA was carried out in order to achieve optimum grafting reaction parameters. Different DG can be easily obtained through adjustment of the above parameters. The characterizations via ATR-FTIR and XPS confirmed the successful graft polymerization of GMA and the introduction of phosphate group onto UHMWPE fibers (Figure 2 and Figure 3).

Screening U(VI) adsorption test showed that the U(VI) adsorption capacity of UHMWPE-g-PO_4_ fiber had a positive correlation with the D_PO_ (Table 1), illustrating that the D_PO_ was a significant factor for extracting uranium [17]. However, a high DG will make the fiber sorbent more brittle, hereby decreasing its mechanical properties [35]. For this reason, fiber with a DG higher than 700% was not used in this work, and the UHMWPE-g-PO_4_ fiber with a DG of 630% (D_PO_: 2.01 mmol∙g^−1^) was selected for the adsorption studies. Batch adsorption experiments showed that the U(VI) adsorption by UHMWPE-g-PO_4_ fiber followed the pseudo-second-order kinetic model and Langmuir isotherm model (Figure 5 and Figure 6). This indicates that the U(VI) adsorption process is a chemisorption process, which is thought to be the monodentate coordination of phosphonyl oxygen and UO_2_^2+^ [36], and the uptake of U(VI) occurs on a homogeneous surface by monolayer adsorption. This result was well consistent with those of phosphate-based mesoporous carbon [21], polyethylene fiber [37], and mesoporous silica [38]. The U(VI) adsorption capacity of UHMWPE-g-PO_4_ fiber (D_PO_: 2.01 mmol∙g^−1^) reached 110.7 mg∙g^−1^ in carbonate solution containing F^−^ ions, indicating its potential application for the efficient removal of U(VI) from alkaline rich effluent or the other contaminated aqueous medium.

For the UHMWPE-g-PO_4_ fiber sorbent, the U(VI) removal ratio was not affected in the presence of Cl^−^, NO_3_^−^, and SO_4_^2^^−^, but significantly reduced with the increasing concentrations of F^−^, H_2_PO_4_^−^, and CO_3_^2^^−^. This might be attributable to that F^−^, H_2_PO_4_^−^, and CO_3_^2^^−^, which have much stronger coordination ability to U(VI) than that of Cl^−^, NO_3_^−^, and SO_4_^2^^−^. Additionally, it can be clearly seen from Figure 7 that (1) the impact of coexisting anions on the U(VI) removal ratio increases in the order of F^−^ < H_2_PO_4_^−^ < CO_3_^2^^−^, (2) H_2_PO_4_^−^ is more prone to coordinate with U(VI) than F^−^ so that UHMWPE-g-PO_4_ fiber is able to extract U(VI) from carbonate solution containing F^−^, and (3) Na_2_CO_3_ aqueous solution is an efficient eluent for the regeneration of UHMWPE-g-PO_4_ fiber for the recycling.

Table 4 shows the U(VI) adsorption capacity of UHMWPE-g-PO_4_ fibers, compared with the other kinds of phosphate-based or phosphonic acid-based adsorbents. The UHMWPE-g-PO_4_ fibers present a good adsorption capacity for extracting U(VI) from a carbonate solution containing F^−^ ions, and can be comparable with those adsorbents with phosphate or phosphonic acid groups [17,20,21,39], extracting U(VI) from aqueous or carbonate solutions without the presence of F^−^ ions. However, the U(VI) adsorption capacity of UHMWPE-g-PO_4_ fibers is lower than those of phosphate-based polyethylene fiber [37] and mesoporous silica [38], and phosphonic acid-based mesoporous silica [40]. This can be mainly attributed to the low initial U(VI) concentration and the existence of F^−^ ions in the carbonate solution. Herein, it should be noted that the fiber sorbents are extremely facile to be placed in U(VI) solution, recovered from solution, and regenerated by eluent, as compared to phosphate-based mesoporous silica and carbon. Furthermore, in comparison with solvent extraction and ion-exchange resin, the UHMWPE-g-PO_4_ fiber sorbent can be directly immersed into the U(VI) solution without the need for auxiliary equipment and the generation of extra waster solution. In addition, the amount of fiber sorbent used can be simply adjusted according to the U(VI) concentration.

Although the selectivity of the UHMWPE-g-PO_4_ fiber for U(VI) is proved to be much higher than for F^−^ in this work, the industrial alkaline effluent usually contains high concentrations of F^−^ (~100 g∙L^−1^) [1], which could drastically reduce the U(VI) adsorption capacity of UHMWPE-g-PO_4_ fiber sorbent. Consequently, future works should be focused on the development of functional polymeric fiber sorbent with enhanced selectivity toward U(VI) in the effluent containing high concentration of F^−^.

## 4. Materials and Methods

### 4.1. Materials

UHMWPE fiber (linear density: 3.6 Denier; diameter: 15 μm) was obtained from Beijing Tongyizhong Specialty Fiber Technology & Development Co., Ltd. GMA (AR), H_3_PO_4_ (GR, ~85 wt%), methanol (CH_3_OH, AR), dichloromethane (CH_2_Cl_2_, AR), UO_2_(NO_3_)_2_∙6H_2_O (B&A Quality), anhydrous Na_2_CO_3_ (AR), anhydrous NaF (AR), anhydrous NaCl (AR), anhydrous NaNO_3_ (AR), anhydrous Na_2_SO_4_ (AR), and NaH_2_PO_4_ (AR) were bought from Sinopharm Chemical Reagent Co., Ltd. (Shanghai, China) All the reagents were directly used without further purification.

### 4.2. Preparation of UHMWPE-g-PO_4_ Fiber Adsorbent

UHMWPE fibers were irradiated in air with an electron beam using 1.5 MeV electrons from a Dynamitron electron beam accelerator (Shanghai Institute of Applied Physics, Chinese Academy of Sciences). The irradiated fibers were immediately immersed in a 100 mL flask containing grafting solutions consisting of GMA in H_2_O/CH_3_OH (50/50 vol%). The flask was then placed in a water bath for grafting under a nitrogen atmosphere. Subsequently, the grafted UHMWPE (UHMWPE-g-PGMA) fibers were thoroughly washed with CH_2_Cl_2_ and water to remove unreacted monomers and homopolymers, and dried at 60 °C overnight under a vacuum. The DG was determined by Equation (5):

DG (%) = (W_g_ − W_o_) × 100/W_o_ (5)

where W_o_ and W_g_ are the weights of the original UHMWPE and UHMWPE-g-PGMA fibers.

The phosphate group was introduced in the fibers through the ring-opening reaction of epoxy groups with H_3_PO_4_. 1 g of UHMWPE-g-PGMA fiber was immersed in 100 mL of H_3_PO_4_ (~85 wt%) at 80 °C for 36 h, in order to drive the reaction to completion [41]. Subsequently, the UHMWPE-g-PO_4_ fibers were washed with deionized water to remove H_3_PO_4_ adhered to the fibers, and dried at 60 °C overnight under a vacuum. The density of phosphate groups (D_PO_, mmol∙g^−1^) was determined by Equation (6):

D_PO_ = (W_PO_ − W_g_) × 1000/(98 × W_PO_) (6)

where W_PO_ is the weight of UHMWPE-g-PO_4_ fibers, and the factor 98 is the molecular weight of H_3_PO_4_.

### 4.3. Characterization

ATR-FTIR spectroscopy was used to characterize the chemical structures of UHMWPE fibers. The spectra were acquired from a Bruker Tensor 27 FT-IR spectrometer, ranging from 600 to 4000 cm^−1^, by averaging 32 scans at a resolution of 4 cm^−1^. The chemical composition of the UHMWPE fibers was measured by XPS, performed with a Thermo SCIENTIFIC ESCALAB 250Xi instrument (Thermo Fisher Scientific Inc., Waltham, MA, USA) using monochromatic Al Kα radiation. The surface morphologies of the graft-modified UHMWPE fibers were observed using SEM (FEI Quanta-250, Hillsboro, OR, USA) under an electron acceleration voltage of 20 kV after sputtering with a thin layer of gold.

### 4.4. U(VI) Sorption Tests

#### 4.4.1. Sorption Kinetics

0.2 g of fiber sorbent was immersed in 1 L of ~20 mg∙L^−1^ U(VI) carbonate solution containing 2 g∙L^−1^ of NaF. The mixture was shaken using a rotary shaker at 25 °C and 100 rpm. 1 mL aliquots were taken from the solution at appropriate time intervals. The U(VI) concentrations for 0, 1, 3, 6, 12, 24, 48, 72, 96, 120, and 144 h in the resulting solutions were analyzed by a Perkin−Elmer Optima 8000 DV inductively coupled plasma optical emission spectrometry (ICP-OES) instrument (PerkinElmer Inc., Waltham, MA, USA). The U(VI) sorption capacity Q_t_ (mg∙g^−1^) and the removal ratio were determined by Equations (7) and (8), respectively:

Q_t_ = (C_0_ − C_t_) × V/m, (7)

removal ratio = (C_0_ − C_t_) × 100/C_0_, (8)

where C_0_ is the initial U(VI) concentration, C_t_ is the U(VI) concentration at various times, V is the volume of solution, and m is the mass of sorbent used.

#### 4.4.2. Sorption Isotherm

A series of U(VI) carbonate solutions containing 2 g∙L^−1^ of NaF were prepared with U(VI) concentrations in the range of 1–30 ppm at pH ~9.6. Sorbent (0.2 g) was added to each solution (1 L), and the trial was carried out for 144 h on a rotary shaker at 25 °C and 100 rpm. The U(VI) concentration was analyzed by ICP-OES. The U(VI) uptake amount Q_e_ (mg∙g^−1^) was calculated from the concentration difference between the beginning and the sorptional equilibrium by Equation (9):

Q_e_ = (C_0_ − C_e_) × V/m (9)

where C_e_ is the U(VI) concentration at equilibrium.

#### 4.4.3. Influence of Coexisting Anions and Sorbent Dosage on U(VI) Removal Ratio

A series of 1 L U(VI) carbonate solutions (~20 mg∙L^−1^) containing various salts were prepared, and aliquots of fiber sorbent (0.2 g) were then added to each solution. In addition, a batch of 1 L U(VI) carbonate solutions (~30 mg∙L^−1^) containing 2 g∙L^−1^ of NaF were prepared, and various dosages of sorbents were then immersed into the solutions. The above mixtures were all shaken using a rotary shaker at 25 °C and 100 rpm. The U(VI) concentrations in the resulting solution before and after adsorption for 144 h were analyzed by ICP-OES. The U(VI) removal ratio was computed by Equation (8).

#### 4.4.4. Recyclability Evaluation

After each adsorption cycle, the fiber sorbent was regenerated by 1 M Na_2_CO_3_ aqueous solution, which can effectively desorb the uranyl ions from the UHMWPE-g-PO_4_ sorbents [21,30]. During the elution, ~0.2 g of the fiber sorbent was immersed in 1 L of Na_2_CO_3_ aqueous solution with continuous shaking at 25 °C and 100 rpm for 24 h. The fiber was then rinsed with deionized water, dried at 60 °C under a vacuum, and used in the next adsorption cycle.

## Figures and Tables

**Table 1:** U(VI) adsorption capacities of UHMWPE-g-PO_4_ fibers with different D_PO_ (initial U(VI) concentrations (C_0_): 20.0 mg∙L^−1^, sorbent: 0.2 g, volume: 1 L, NaF: 2.0 g∙L^−1^, time: 24 h, and temperature: 25 °C).

Sorbents	Sorbent Description		Adsorption Capacity
	DG (%)	D_PO_ (mmol∙g^−1^)	U(VI) (mg∙g^−1^)
A	186	1.55	38.9 ± 1.1
B	294	1.81	45.3 ± 0.7
C	540	1.93	56.4 ± 1.3
D	630	2.01	69.2 ± 2.0

**Table 2:** Kinetic parameters for the sorption of U(VI) by UHMWPE-g-PO_4_ fiber.

Pseudo-First-Order			Pseudo-Second-Order		
Q_e_ (mg∙g^−1^)	*k*_1_ (h^−1^)	R^2^	Q_e_ (mg∙g^−1^)	*k*_2_ (g∙mg^−1^∙h^−1^)	R^2^
63.1	3.17 × 10^−2^	0.978	95.2	1.48 × 10^−3^	0.999

**Table 3:** Parameters calculated from Langmuir and Freundlich models for the capture of U(VI) by UHMWPE-g-PO_4_ fiber.

Langmuir Model			Freundlich Model		
Q_max_ (mg∙g^−1^)	*K*_L_ (L∙mg^−1^)	R^2^	*K*_F_ (mg∙g^−1^)	*n*	R^2^
117.1	2.0	0.999	79.1	5.7	0.776

**Table 4:** U(VI) adsorption performance of UHMWPE-g-PO_4_ fibers compared with other adsorbents containing phosphate or phosphonic acid groups.

Sorbents	C_0_ (mg∙L^−1^)	pH	CO_3_^2−^	F^−^	Q_max_ (mg∙g^−1^)	Reference
phosphonic acid-based mesoporous silica	8	8.3	with	without	54.5	[17]
vinylphosphonic acid grafted poly(vinyl alcohol) fiber	98.6	8.0	without	without	30.0	[20]
Phosphate-based mesoporous carbon	50	8.0	without	without	70.0	[21]
phosphate-based polyethylene fibers	50	8.2	without	without	151.0	[37]
phosphate-based mesoporous silica	160	6.9	without	without	303.0	[38]
phosphonate-based polystyrene microsphere	200	8.0	with	without	83.4	[39]
phosphonic acid-based mesoporous silica	42.8	8.0	without	without	207.6	[40]
phosphate-based UHMWPE fiber	20	9.6	with	with	110.7	this work
